# The association between 24-h blood pressure variability and major adverse cardiac events (MACE) in hospitalized patients with acute myocardial infarction: a retrospective cohort study

**DOI:** 10.1186/s43044-021-00213-1

**Published:** 2021-10-14

**Authors:** Harefa, Ika Prasetya Wijaya, Muhadi, Cleopas Martin Rumende, Sally Aman Nasution, Sukamto Koesnoe, Maruhum Bonar Marbun, Hamzah Shatri

**Affiliations:** Department of Internal Medicine, Faculty of Medicine, University of Indonesia/Dr, CiptoMangunkusumo National Hospital Jakarta, Jakarta, Indonesia

**Keywords:** Acute myocardial infarction, Blood pressure variability, Average real variability, Major adverse cardiac events

## Abstract

**Background:**

Acute myocardial infarction (AMI) is major cardiovascular disease that causes high morbidity and mortality. In AMI, ischemia and necrosis affected some cardiomyocytes leading to a decrease in myocardial contractility which is followed by an acute proinflammation reaction and increased sympathetic tone. Meanwhile, high blood pressure variability (BPV) causing an increased left ventricular workload, heart rate, myocardial oxygen demand and induces proinflamations and endothelial dysfunction. Therefore a high BPV and its associated pathological effects are likely to aggravate the physiological function of the heart and affect the emergence of acute cardiac complications in AMI patients. This study aims to investigate the association’s between short-term BPV and major adverse cardiac events (MACE) in AMI patients. This retrospective cohort study used simple random sampling to identify AMI patients who were hospitalized at Cipto Mangunkusumo National Hospital between January 2018 and December 2019. Mann Withney was performed to investigate the association between BPV and MACE.

**Results:**

The average systolic BPV value which was calculated as standard deviation (SD) and average real variability (ARV) was higher in the MACE group than in the non-MACE group. Systolic SD and systolic ARV in the MACE group were 13.28 ± 5.41 mmHg and 9.88 ± 3.81 mmHg respectively. In the non-MACE group, systolic SD and systolic ARV were 10.76 (4.59–26.17) mmHg and 8.65 (3.22–19.35) mmHg respectively. There was no significant association between BPV and MACE. However, there were significant differences between systolic SD and systolic ARV in patients with hypertension who experienced MACE and patients without hypertension who experienced MACE.

**Conclusions:**

The BPV of AMI patients who experience MACE was higher than that of non-MACE AMI patients. There was no significant association between BPV ​​and MACE during the acute phase of AMI.

**Supplementary Information:**

The online version contains supplementary material available at 10.1186/s43044-021-00213-1.

## Background

Acute myocardial infarction (AMI) is a major cardiovascular disease that causes morbidity and mortality. The clinical picture of myocardial cell death in AMI includes abnormal cardiac biomarker levels accompanied by clinical signs of ischemia, such as chest pain and electrocardiogram feature changes. Ischemic myocardial cells trigger various cellular, hormonal and immune responses in the heart. These responses include the release of proinflammatory cytokines and the activation of cellular and hormonal immunity, which in turn will initiate the process of necrosis, apoptosis, autophagia, and proinflammatory reactions in the heart. In the ischemic area, oxidative stress builds up, and myocardial cell’s ability to produce vasodilator substances decreases. Mechanically, some injured cardiomyocytes leading to a decrease in myocardial contractility, which is compensated by an increase in sympathetic activity and the secretion of catecholamines as well as angiotensinogen, which aims to maintain peripheral hemostasis function [1–3].

Most AMI patients have a history of endothelial dysfunction with manifestations of atherosclerosis in the blood vessels and coronary arteries. Atherosclerosis is a chronic inflammatory condition characterized by high levels of mediators and proinflammatory cytokines in the systemic system. Endothelial dysfunction occurs when the balance between vasodilator and vasoconstrictor components is disturbed in the form of the decreased production and bioavailability of nitric oxide (NO) as well as increased levels of oxidative stress. Endothelial dysfunction and atherosclerosis also lead to baroreflex dysfunction. Decreased baroreflex function, sympathetic hyperactivity, and high systemic vasoconstrictor levels result in blood pressure lability, high blood pressure variability (BPV), and proarrhythmic conditions. Hypersympathetic conditions also tend to increase the heart rate which increases the heart’s oxygen demand. In addition, a proinflammatory state, atherosclerosis, and endothelial dysfunction put AMI patients in a prothrombotic state which is characterized by high platelet activity and erythrocyte aggregation in the circulatory system [4–6].

BPV is a hemodynamic component that exerts shear stress and circumferential stretch on the endothelial and vessel intima, which can trigger a series of biological and cellular responses in the vessels. Disturbance in the balance of biological and cellular responses will result in impaired function of the endothelium and the coronary arteries as a whole. High BPV will have biological and mechanical consequences on the heart in the form of increased workload and myocardial oxygen demand. This effect is further amplified by the lability and fluctuation of blood pressure in infarcted patients due to sympathetic hyperactivation and baroreflex dysfunction. Vascular abnormalities, inflammation, sympathetic hyperactivation, and autonomic nervous system dysfunction greatly increase the damaging effects of BPV on target organs. Such, the pathological processes affected by to high BPV could be aggravating mechanical and biological heart functions in AMI patients and cause major adverse cardiac events (MACE) during the acute phase of AMI [4–6].

## Methods

### Study subjects and sample size

The sample population was comprised of all AMI patients who were hospitalized between January 2018 and December 2019. The simple random sampling method was used to select participants for the present study.

### Study design and procedure

This retrospective cohort study was approved by the medical research ethics committee of Cipto Mangunkusumo National Hospital. Patients on mechanical ventilation; patients with active inflammatory diseases, such as infections or malignancies; patients with incomplete medical records, patients with MACE on admission, and patients with severe valve abnormalities were excluded from the study. AMI was defined according to the 2017 European Society of Cardiology definition, and the MACE criteria were malignant arrhythmia, acute heart failure/acute pulmonary edema, cardiogenic shock, and cardiac death within the first five days of hospitalization.

Systolic and diastolic blood pressure was automatically recorded with an oscillometer every 60 minutes for the first 24 hours. Patients were checked for an occurrence of MACE every day for the first five days of hospitalization according to a medical record. The standard deviation (SD) and the average real variability (ARV) of BPV were calculated according to the following formulas.

$$SD = \sqrt {\frac{1}{N - 1}} \sum\limits_{(k = 1)}^{N} {(TD_k + 1 - TD\;k)^{2} }$$

$$ARV = \frac{1}{N - 1}\sum\limits_{k = 1}^{n - 1} {(TD_{(k + 1)} - TD\;k)\quad \text{k} = 1}$$

### Data analysis

Description and data analysis was performed using a *software platform offers advanced statistical analysis* (SPSS) v28. Results were considered significant at α<0.05 and the 95% confidence interval (CI). The Shapiro Wilk test was conducted to test the normality distribution of the data. Mean ± SD was used for data with a normal distribution, while the median (highest-lowest value) ware used for data that was not normally distributed. The Mann-Whitney test was used to determine the relationship between BPV and the incidence of MACE in AMI patients, and multivariate analysis was used to assess the effect of risk factors on the incidence of MACE.

## Results

Between January 2018 and December 2019, 357 AMI patients with ST-elevation myocardial infarction (STEMI) and non-ST-elevation myocardial infarction (NSTEMI) were hospitalized at Cipto Mangunkusumo National Hospital. Seventeen patients were excluded because they did not meet the study criteria. A total of 340 patients were randomized to get 120 participants.

Of the 120 patients in the sample population, 62.5% had STEMI and 37.5% had NSTEMI. Most of the AMI patients were male, with a mean age of 57.13 ± 10.53 years. The youngest patient was 29 years old and the oldest was 89 years old. Hypertension is the most common risk factor, followed by dyslipidemia, smoking, diabetes mellitus, and chronic kidney disease. MACE was obtained in 19 patients consisting of eight patients with malignant arrhythmias, five patients with acute heart failure, three patients with cardiac arrest, and three patients with cardiogenic shock (Table 1).

**Table 1:** AMI patients characteristics

Variable	N = 120
Sex, n (%)	
Male	90 (75.0)
Female	30 (25.0)
Age (year), Mean (SD)	57.13 ± 10.53
Body Mass Index, Median (range)	24.18 (22.22–27.53)
Diabetes mellitus, n (%)	52 (43.3)
Hypertension, n (%)	92 (76.7)
Dyslipidemia, n (%)	76 (63.3)
Smoking, n (%)	74 (61.7)
Chronic Kidney Disease, n (%)	30 (25.0)
Previous Cardiovascular Disease, n (%)	31 (25.9)
MACE, n (%)	19 (15.8)
AMI STEMI / NSTEMI	75 (62.5) / 45 (37.5)
Systolic blood pressure	117.83 ± 16.02
Diastolic blood pressure	76.64 ± 9.88
SD-SBP	11.98 ± 4.68
SD-DBP	8.82 ± 3.63
ARV-SBP	9.36 ± 3.48
ARV-DBP	7.73 ± 2.98

SD-SBP = standard deviasion of systolic blood pressure; SD-DBP = standard deviasion of diastolic blood pressure

ARV-SBP = average real variability of Systolic blood pressure; ARV-DBP = average real variability of diastolic blood pressure; MACE = Major Adverse Cardiac Evens; AMI = acute myocardial infarct

STEMI = acute myocardial infarction with ST segment elevation

NSTEMI = acute myocardial infarction without ST segment elevation

The mean of 24-hours systolic and diastolic blood pressure values were 117.83 ± 16.02 mmHg and 76.64 ± 9.88 mmHg, respectively. The 24-hours systolic-diastolic BPV (SD-BPV/SD-DBP) and the systolic-diastolic ARV (ARV-SBP/ARV-DBP) were 11.98 ± 4.68 mmHg, 8.82 ± 3.63 mmHg, 9.36 ± 3.48 mmHg, 7.73 ± 2.98 mmHg respectively (Table 2). The mean value of systolic BPV in the group with MACE (SD-SBP and ARV-SBP) was higher than the group without MACE. However, there was no significant relationship between VTD and the incidence of MACE. (p>0.05)

**Table 2:** Relationship between BPV and MACE*

Variable	MACE	Non-MACE	Z-value	p-value
SD-SBP	13.28 ± 5.14	10.76 994.59–26.17)	− 1.459	0.144
SD-DBP	7.69 (4.77–21.90)	7.99 (3.23–24.57)	− 0.151	0.880
ARV-SBP	9.88 ± 3.81	8.65 (3.22–19.35)	− 0.554	0.580
ARV-DBP	6.87 (3.87–16.43)	7.04 (3.26–17.13)	− 0.025	0.980

SD-SBP = standard deviation of systolic blood pressure; SD-DBP = standard deviation of diastolic blood pressure

ARV-SBP = average real variability of Systolic blood pressure; ARV-DBP = average real variability diastolic blood pressure

MACE = major adverse cardiac evens; BPV = blood pressure variability

As shown in Table 3, the mean BPV in AMI patients with hypertension who experienced MACE was significantly higher than in patients without hypertension who experienced MACE (p<0.05).

**Table 3:** BPV in patients MACE and hypertension and patients with MACE without hypertension *

BPV	Hypertension		*P* value
	Yes (n = 18)	No (n = 1)	
SD SBP, Median (Range)	13.13 (10.89–15.97)	8.14	**0.013**
SD DBP, Median (Range)	7.75 (6.38–11.19)	6.65	0.315
ARV SBP, Median (Range)	9.89 (6.76–13.33)	5.91	**0.035**
ARV DBP, Median (Range)	7.00 (5.68–9.59)	6.35	0.400

^*^Mann-Withney test

SD-SBP = standard deviation of systolic blood pressure; SD-DBP = standard deviation of diastolic blood pressure

ARV-SBP = average real variability of Systolic blood pressure; ARV-DBP = average real variability diastolic blood pressure

BPV = blood pressure variability; MACE = major adverse cardiac events

Bold font mean a difference was statistically significant (*p* < 0.05)

A multivariate analysis was done to determine the most important risk factors for MACE in AMI patients (Additonal file 1: Table 4). The variables that influence the incidence of MACE are hypertension, smoking and a history of cardiovascular disease (CVD). The strength of the relationship was hypertension (OR = 7.452), smoking (OR = 3.902) and CVD (OR = 2.832).

## Discussion

The high incidence of AMI in older and males may be due to an increased incidence of metabolic diseases, such as hypertension and diabetes as well as smoking in men. Indonesian health survey in 2018 found that 10.9% and 34.1% of the population had diabetes mellitus and hypertension respectively, and that these percentages increase with age. For example, the prevalence of hypertension was 45.3% among 45–54 years old and increased to 63.2% among 65–74 years old. In addition, 31.3% of the hypertension sufferers were male. Meanwhile, the World Health Organisation (2015) show that 24.1% of male and 20.1% of female worldwide had high blood pressure. There was an increase in the number of hypertensive patients from 594 million in 1975 to more than 1.13 billion in 2015 [7, 8].

Birry K et al. (2012) found that the mean age of acute coronary syndromes (ACS) patients ware 56.24 ± 11.18 years, and 67.3% were male; these demographics are almost the same as those in the present study. Similarly, in Zulkifli et al. (2017) study, the mean age of ACS patients was 59.97 ± 10.62 years, and 72.5% were male [9, 11].

The incidence of MACE in this study was 15.8%. This result is higher than that of previous studies conducted at RSCM. Diah et al. (2018) and Anastasia et al. (2019) conducted studies on ACS patient populations and found that 9.6% and 11.9%, respectively had MACE. Diah found 25%, 36.5%, and 38% of patients had unstable angina pectoris, STEMI, and NSTEMI, respectively. For the same conditions, Anastasia's results were 37.5%, 37.5%, and 25%, respectively. In the present study, which only focused on AMI patients with STEMI or STEMI, 62.5% had STEMI, and 37.5% had NSTEMI. The large proportion of patients with STEMI led to a higher prevalence of MACE in the sample population because, as shown in the literature, the risk of MACE in the acute period of STEMI is much greater than in NSTEMI or unstable angina pectoris [9–11].

The average BPV value in the present study was slightly lower than the value identified by Mancia (2007), who studied non-AMI patients who died from cardiovascular events. The mean SD-SBP and SD-DBP in Mancia’s study were15.3 ± 3.9 mmHg and 12.7 ± 3.4 mmHg, respectively. Meanwhile, systolic and diastolic ARV was 8.6 ± 1.4 mmHg and 9.9 ± 1.6 mmHg, respectively. This difference between Mancia’s and the present study may be due to close monitoring of BP and immediate pharmacological intervention in the intensive care unit. In contrast, BPV assessments of people who are not hospitalized, the control and supervision of BP is highly dependent on individual awareness [12, 13].

Hassan et al. (2017), conducted a prospective cohort study with 200 samples and found a significant correlation between a high BPV and the incidence of MACE in AMI patients who were observed for the first seven days of hospitalization. In Hassan’s study, a weighted standard deviation of blood pressure (wSD-BP) and the standard deviation of the 24-hour systolic-diastolic blood pressure (SD-SBP/SD-DBP) was used to measure BPV. Hassan et al. found a significant correlation between the high wSD-BP group (>12.6mmHg) and the high SD-SBP group (>13.5 mmHg) and the incidence of MACE (r=0.56, *p*=0.003). The present study findings indicate that BPV and MACE did not have a significant relationship in the first five days of AMI patient hospitalization. However, the average BPV value in the MACE group was higher than in the non-MACE group. The result is in line with the finding in Hassan et al study [14].

The non-significance relationship between BPV and MACE found in the present study could be due to the study population, which included patients with various cardiovascular risk factors. The BPV characteristics in each comorbid disease affected the average BPV of the entire study population and affected the relationship between BPV and MACE.

Variations in blood pressure mainly cause mechanical responses in the form of shear stress and circular stretch on the endothelium and blood vessel walls. These pressure and stretch forces cause deformity of the vessel structure consisting of cells and connective tissue, which in turn, stimulate the release of biochemical reactants. On physiological conditions, there is a balance between mechanical and chemical stimuli with biological responses which maintain the hemostatic function of blood vessels. In vitro studies have shown that an increased acute stress load immediately induces signaling sequences and remodeling of the endothelium. This series of responses include activation of nuclear factor-κβ (NF-κβ), intercellular adhesion molecules-1 (ICAM-1), monocyte chemoattractant protein-1 (MCP-1), tissue factor, platelet-derived growth factor-B (PDGF-B), transforming growth factor (TGF-β1), cyclooxygenase-2 (COX-2), endothelial nitric oxide synthase (eNOS) and manganese superoxide dismutase. The aforementioned biological processes lead to the release of NO and prostacyclin, which are the main vessels vasodilators [15, 16].

Regarding mechanobiological response, chronic stress and strain decrease the production of eNOS, endothelin-1(ET-1), and PDGF-B. In addition, lead to a decrease in the production of prostaglandin vasodilators and an increase in oxidative stress production, the activation of platelet and erythrocyte aggregation, and cell hypertrophy as well as an increase in the density of actin-myosin filaments. The mechanobiological response above predisposes patients to atherosclerosis, prothrombotic conditions, and chronic systemic inflammation, which in turn have a pathological effect on the cardiovascular system and future MACE risk [15, 16].

The non-significant relationship between BPV and MACE in the presents study may also be because the stress and strain loading caused by BPV tends to lead to chronic low-grade inflammation response. Traditional factors, such as the location and extent of the infarct, the number and location of the coronary vessels involved, and the functional capacity of the heart before the attack, seem to be the primary determinants of the incidence of MACE during the acute period of AMI. According to the study finding, the acute inflammation that occurs in AMI which is exacerbated by the vascular inflammatory process in response to pressure and strain loads, is not strong enough to induce MACE in AMI. Duplication of the inflammatory response due to AMI, increase ventricular load and myocardial oxygen demand caused by high BPV that could be associated with the incidence of MACE in the acute period of AMI, was not supported.

As previously mentioned, the systolic BPV in AMI patients with hypertension who experienced MACE was significantly higher than in patients without hypertension who experienced MACE. This was probably caused by the underlying disease suffered by the patients. Previous studies have shown that BPV tends to be higher in the hypertensive population than in the non-hypertensive population. The occurrence of AMI followed by an increased sympathetic activity and baroreceptor dysfunction further increase BPV in the hypertensive population [17–19].

Furthermore, high BPV in hypertensive patients is amplified by increased sympathetic activity and heart rate during AMI. This pathological condition is further exacerbated by the high sympathetic activity, vasoactive-vasodilator imbalance, renin-angiotensin aldosterone system activation, and chronic inflammation that was already present in hypertensive patients. A high BPV value and the accompanying pathological consequences are likely to have a large resultant effect on target organs and the emergence incidence of MACE in hypertensive AMI patients [17, 18].

The effect of hypertension on the incidence of MACE was further strengthened by the results of the multivariate analysis conducted on existing cardiovascular risk factors. It was found that the following variables influence the incidence of MACE; hypertension (OR=7.452), smoking (OR=3.902), and previous cardiovascular disease (OR=2.832). The significant difference in BPV values in patients with hypertension who experience MACE compared with patients without hypertension who experience MACE illustrates the important role of BPV in hypertensive patients in terms of MACE complications during AMI. The identification of hypertension as a major risk factor for MACE raises questions for further research on the effect of BPV on the incidence of MACE in patients with hypertension.

As a retrospective study, technical errors in BP measurement for some patients cannot be avoided. To the best of our’s knowledge, this is the first study in Indonesia to investigate the effect of BPV on the incidence of MACE. As such, we hope that this study will increase literacy about BPV and its relation to MACE and improve standard management for AMI patients.

## Conclusions

The BPV of AMI patients who experience MACE was higher than that of non-MACE AMI patients and the SD-SBP and ARV-SBP of AMI patients with hypertension who experienced MACE ware significantly higher than that of AMI patients without hypertension who had MACE. Overall there was no significant association between BPV and MACE in AMI patients.

## Limitation

In this retrospective cohort study, measurement errors due to technical problems such as cuff size, cuff position shift and so on that may occur in some patients cannot be ruled out. In addition, the pressure data obtained are data recorded by the monitoring unit which should be calibrated repeatedly before, during the study with a mercury sphymomanometer, to assess the level of accuracy.

## Supplementary Information

**Additional file 1: Table 4.** Risk Factors for MACE in AMI patients.

## Data Availability

Data is available on request.

## Abbreviations

AMI: Acute Myocardial Infarction
BPV: Blood Pressure Variability
MACE: Major Adverse Cardiac Evens; AMI = acute myocardial infarct
STEMI: Acute myocardial infarction with ST-segment elevation
NSTEMI: Acute myocardial infarction without ST-segment elevation
SD-SBP: Standard deviation of systolic blood pressure
SD-DBP: Standard deviation of diastolic blood pressure
ARV-SBP: Average real variability of Systolic blood pressure
ARV-DBP: Average real variability of diastolic blood pressure
