# Supplementary material for: The association between 24-h blood pressure variability and major adverse cardiac events (MACE) in hospitalized patients with acute myocardial infarction: a retrospective cohort study
Source: Egypt Heart J. 2021 Oct 14;73:88. doi: 10.1186/s43044-021-00213-1 (PMC8517047; doi:10.1186/s43044-021-00213-1)
Supplement: Supplementary file 1 — Additional file 1: Table 4. Risk Factors for MACE in AMI patients. [file 43044_2021_213_MOESM1_ESM.docx]

| Table 4. Risk Factors for MACE in AMI Patients | | | | | | | | | |
| --- | --- | --- | --- | --- | --- | --- | --- | --- | --- |
|  | | B | S.E. | Wald | df | Sig. | Exp(B) | 95% C.I.for EXP(B) | |
|  |  |  |  |  |  |  |  | Lower | Upper |
| Step 1^a^ | MCI(1) | .518 | .599 | .747 | 1 | .387 | 1.679 | .519 | 5.436 |
|  | DM(1) | .385 | .578 | .443 | 1 | .506 | 1.469 | .473 | 4.564 |
|  | HT(1) | 1.961 | 1.092 | 3.226 | 1 | .072 | 7.105 | .836 | 60.377 |
|  | DIS(1) | .287 | .604 | .225 | 1 | .635 | 1.332 | .408 | 4.353 |
|  | SMOKING(1) | 2.063 | .898 | 5.277 | 1 | .022 | 7.870 | 1.354 | 45.757 |
|  | CKD(1) | .558 | .620 | .809 | 1 | .368 | 1.747 | .518 | 5.888 |
|  | CVD(1) | 1.089 | .585 | 3.470 | 1 | .062 | 2.971 | .945 | 9.344 |
|  | Sex(1) | -.877 | .882 | .987 | 1 | .320 | .416 | .074 | 2.346 |
|  | Constant | -5.327 | 1.444 | 13.607 | 1 | .000 | .005 |  |  |
| Step 2^a^ | MCI(1) | .517 | .599 | .745 | 1 | .388 | 1.677 | .519 | 5.421 |
|  | DM(1) | .429 | .570 | .565 | 1 | .452 | 1.535 | .502 | 4.692 |
|  | HT(1) | 1.975 | 1.092 | 3.274 | 1 | .070 | 7.210 | .848 | 61.271 |
|  | SMOKING(1) | 2.016 | .882 | 5.221 | 1 | .022 | 7.507 | 1.332 | 42.308 |
|  | CKD(1) | .623 | .605 | 1.061 | 1 | .303 | 1.865 | .570 | 6.108 |
|  | CVD(1) | 1.068 | .581 | 3.377 | 1 | .066 | 2.909 | .931 | 9.085 |
|  | Sex(1) | -.834 | .869 | .919 | 1 | .338 | .434 | .079 | 2.388 |
|  | Constant | -5.184 | 1.398 | 13.750 | 1 | .000 | .006 |  |  |
| Step 3^a^ | MCI(1) | .412 | .577 | .509 | 1 | .476 | 1.509 | .487 | 4.677 |
|  | HT(1) | 1.986 | 1.093 | 3.299 | 1 | .069 | 7.284 | .855 | 62.079 |
|  | SMOKING(1) | 1.930 | .881 | 4.801 | 1 | .028 | 6.893 | 1.226 | 38.757 |
|  | CKD(1) | .652 | .601 | 1.180 | 1 | .277 | 1.920 | .592 | 6.232 |
|  | CVD(1) | 1.066 | .577 | 3.413 | 1 | .065 | 2.904 | .937 | 8.999 |
|  | Sex(1) | -.825 | .874 | .892 | 1 | .345 | .438 | .079 | 2.429 |
|  | Constant | -4.895 | 1.335 | 13.436 | 1 | .000 | .007 |  |  |
| Step 4^a^ | HT(1) | 1.955 | 1.088 | 3.229 | 1 | .072 | 7.061 | .838 | 59.531 |
|  | SMOKING(1) | 1.864 | .855 | 4.751 | 1 | .029 | 6.449 | 1.207 | 34.464 |
|  | CKD(1) | .559 | .583 | .917 | 1 | .338 | 1.748 | .557 | 5.485 |
|  | CVD(1) | 1.035 | .576 | 3.229 | 1 | .072 | 2.816 | .910 | 8.711 |
|  | Sex(1) | -.732 | .846 | .750 | 1 | .387 | .481 | .092 | 2.523 |
|  | Constant | -4.597 | 1.259 | 13.322 | 1 | .000 | .010 |  |  |
| Step 5^a^ | HT(1) | 1.916 | 1.076 | 3.167 | 1 | .075 | 6.791 | .823 | 56.007 |
|  | SMOKING(1) | 1.417 | .641 | 4.879 | 1 | .027 | 4.123 | 1.173 | 14.489 |
|  | CKD(1) | .575 | .580 | .981 | 1 | .322 | 1.777 | .570 | 5.542 |
|  | CVD(1) | .963 | .571 | 2.844 | 1 | .092 | 2.619 | .855 | 8.018 |
|  | Constant | -4.783 | 1.233 | 15.041 | 1 | .000 | .008 |  |  |
| Step 6^a^ | HT(1) | 2.008 | 1.070 | 3.524 | 1 | .061 | 7.452 | .915 | 60.677 |
|  | SMOKING(1) | 1.362 | .638 | 4.551 | 1 | .033 | 3.902 | 1.117 | 13.633 |
|  | CVD(1) | 1.041 | .565 | 3.400 | 1 | .065 | 2.832 | .937 | 8.565 |
|  | Constant | -4.685 | 1.226 | 14.592 | 1 | .000 | .009 |  |  |
| A multivariate analysis to determine the most important risk factors for MACE in AMI patients. The variables that influence the incidence of MACE are hypertension, smoking and previous history of cardiovascular disease. The strength of the relationship was hypertension (OR=7.452), smoking (OR=3.902) and CVD (OR=2.832).  MCI=acute myocardial infarction; HT=Hypertension;DM=diabetes mellitus;CKD=chronic kidney disease;CVD=previous cardiovascular disease. | | | | | | | | | |
